# Effect of childhood socioeconomic conditions on cancer onset in later life: an ambidirectional cohort study

**DOI:** 10.1007/s00038-018-1111-9

**Published:** 2018-05-17

**Authors:** Bernadette W. A. van der Linden, Delphine S. Courvoisier, Boris Cheval, Stefan Sieber, Piet Bracke, Idris Guessous, Claudine Burton-Jeangros, Matthias Kliegel, Stéphane Cullati

**Affiliations:** 1 Swiss NCCR “LIVES - Overcoming Vulnerability: Life Course Perspectives”, University of Geneva, Geneva, Switzerland; 2 Center for the Interdisciplinary Study of Gerontology and Vulnerability (CIGEV), University of Geneva, 28 Boulevard du Pont d’Arve, 1205 Geneva, Switzerland; 3 Department of General Internal Medicine, Rehabilitation and Geriatrics, University of Geneva, Geneva, Switzerland; 4 Department of Sociology, Ghent University, Ghent, Belgium; 5 Unit of Population Epidemiology, Department of Community Medicine, Primary Care and Emergency Medicine, Geneva University Hospitals, Geneva, Switzerland; 6 Department of Epidemiology, Emory University, Atlanta, GA USA; 7 Institute of Social and Preventive Medicine, Lausanne University Hospital, Lausanne, Switzerland; 8 Department of Ambulatory Care and Community Medicine, University of Lausanne, Lausanne, Switzerland

**Keywords:** Cancer, Socioeconomic conditions, Life course, Old age, Ageing

## Abstract

**Objectives:**

Living in low socioeconomic conditions during childhood is associated with poor health outcomes in later life. Whether this link also applies to cancer is unclear. We examined whether childhood socioeconomic conditions (CSCs) are associated with cancer risk in later life and whether this effect remained after adjusting for adulthood socioeconomic conditions (ASCs).

**Methods:**

Data for 26,431 individuals ≥ 50 years old included in SHARE were analysed. CSCs were constructed by using indicators of living conditions at age 10. ASC indicators were education, main occupation, and household income. Gender-stratified associations of CSCs with cancer onset (overall and by site) were assessed by Cox regression.

**Results:**

In total, 2852 individuals were diagnosed with cancer. For both men and women, risk of overall cancer was increased for advantaged CSCs and remained so after adjusting for ASCs (hazard ratio = 1.36, 95% CI 1.10, 1.63, and 1.70, 95% CI 1.41, 2.07).

**Conclusions:**

Advantaged CSCs are associated with an increased risk of overall cancer at older age, but results vary by cancer sites and sex. Participation in cancer screening or exposure to risk factors may differ by social conditions.

**Electronic supplementary material:**

The online version of this article (10.1007/s00038-018-1111-9) contains supplementary material, which is available to authorized users.

## Introduction

Socioeconomic differences in health exist and can also be seen in cancer incidence, prevalence, and survival across populations. Studies found an association between lower socioeconomic status and higher incidence of respiratory, oesophagus, stomach, and cervical cancers, and higher socioeconomic status and colon, breast, and ovary cancers, and skin melanoma, with overall better survival in patients from higher socioeconomic status (Bouchardy et al. 2006; Faggiano et al. 1997; Weiderpass and Pukkala 2006). These differences are thought to be largely attributed to aetiological factors, such as diet, physical activity, and smoking, but part of the variation remains unexplained (Weiderpass and Pukkala 2006). Given the complex aetiology of cancer and often long latency period, adopting a life course perspective helps to better understand the different pathways that may affect cancer onset in later life and thus explain more of the variation (Potischman et al. 2004).

From a life course perspective, three theories on the relation between socioeconomic conditions and cancer onset in later life can be considered. First of all, during developmental processes, biological systems are more sensitive to external influences (Bruer 2001). These sensitive periods take place mainly in early life, as this is the time in which most developmental processes occur. Recent research focused on cancer suggested that stressful conditions and adverse events in early life such as trauma, abuse, or maltreatment increase the risk of developing cancer in adulthood (Kelly-Irving et al. 2013a, b). A second theory refers to cumulative (dis)advantage, considering that (dis)advantage in early life leads to an accumulation of subsequent (dis)advantages (Dannefer 2003). The third theory is related to social mobility, suggesting that risk associated with childhood disadvantage could be decreased or partially compensated for individuals moving from low childhood socioeconomic status to a higher status in adulthood (Luo and Waite 2005).

Taking into account both childhood and adulthood socioeconomic conditions (ASCs) could give suggestions on whether childhood socioeconomic conditions (CSCs) have a long-lasting effect on cancer in later life, beyond ASCs, i.e. health inequalities would be related to both CSCs and ASCs. Alternatively, it allows testing whether CSCs channel individuals into life course trajectories leading to social destinations or pathways, thus suggesting that CSCs are the actual determinant of health at older age, over ASCs (i.e. there is no longer an association between CSCs and cancer in later life once adjusting for ASCs) (Hertzman and Power 2003). Additionally, it is known that some risk factors for site-specific cancers are more closely related to adulthood. For example, one study showed that mortality from stomach cancer was dependent on CSCs, whereas mortality from lung cancer was mainly dependent on adulthood factors (Smith et al. 1998). This suggests that low socioeconomic conditions in different life stages may be related to risk of different site-specific cancers.

A recent review on CSCs and adult cancer identified only two studies that investigated both the independent and joint effect of CSCs and ASCs on cancer incidence (Vohra et al. 2016). The first study, looking at breast cancer incidence and survival, found an increased breast cancer incidence for a higher level of mothers’ education and family income in early life (Pudrovska and Anikputa 2012). The effect of mothers’ education was mediated by women’s socioeconomic status in adulthood and reproductive behaviour. Education of the father was negatively related to breast cancer survival, and this effect was further mediated by women’s education. The second study found an association between low CSCs and higher risk of colorectal cancer and reduced risk of basal cell carcinoma, which remained significant after adjusting for ASCs. Conversely, no associations between CSCs and total cancer, lung, breast, and prostate cancer were found (de Kok et al. 2008).

To sum up, the review on the relationship of poor CSCs and cancer later in life, finds overall weak and inconsistent evidence in terms of the direction of the effect (Vohra et al. 2016). Additionally, evidence on both direct and indirect effects via possible ASCs mediating pathways is scarce. Since there are only a few studies with heterogeneous results, the first aim of this study was to examine whether CSCs are associated with cancer onset in later life by using longitudinal data for older adults from 14 countries across Europe. The second aim was to test whether this effect remained after adjusting for ASCs, for both cancer overall and by site.

## Methods

### Study design and population

Data for 26,431 individuals were retrieved from the Survey of Health, Ageing, and Retirement in Europe (SHARE) database. SHARE is a longitudinal, cross-national, and ambidirectional survey designed to investigate population ageing processes and includes data for individuals ≥ 50 years old (Borsch-Supan et al. 2013). SHARE includes six waves of data, collected every 2 years between 2004 and 2016. Participants were eligible for the current analyses if they participated in the third wave and at least one other wave. Participation in the third wave was a prerequisite since retrospective life course information related to socioeconomic conditions was collected in this wave (SHARELIFE). Any other wave was used to collect information on cancer cases. The duration of follow-up was 12 years at maximum, but it was not equal for all participants as some did not participate the whole time. Participants were from 14 European countries—Austria, Belgium, Czech Republic, Denmark, France, Germany, Greece, Ireland, Italy, the Netherlands, Poland, Spain, Sweden, and Switzerland—based on probability sampling adapted to each country. SHARE was approved by the relevant research ethics committees in the participating countries, and all participants provided written informed consent.

### Cancer

Cancer was operationalized by using the SHARE question “Has a doctor ever told you that you had/Do you currently have any of the conditions on this card?” (de Souto Barreto et al. 2017). It was specified that a doctor had told the participants that they currently have this condition or that they were treated for or were affected by this condition. If participants selected the option “Cancer: ever diagnosed/currently having”, they were included in the analyses as having cancer. Additionally, the question on the individual’s age at diagnosis was used to determine when the cancer was diagnosed, which can be before follow-up in the study. The follow-up question on specific cancer sites was used for analyses by site. These questions were asked at every wave except wave three. Only the first diagnosis was taken into consideration since the event is first cancer diagnosis.

### Childhood socioeconomic conditions

The variable CSCs were determined by using the measure of childhood circumstances by Wahrendorf and Blane (Wahrendorf and Blane 2015). It was constructed by combining four binary indicators of socioeconomic conditions at age 10 that are relevant when assessing the long-term effects of early life socioeconomic conditions on health; the occupational position of the main breadwinner in the household, the number of books at home, overcrowding in the household, and housing quality (Dedman et al. 2001; Evans et al. 2010) (see Online Resource 1 for details). These variables were measured as part of the retrospective SHARELIFE module in wave 3.

### Covariates, confounders, and mediators

All analyses were adjusted for attrition (no dropout, dropped out, deceased) and for potential confounders, including birth cohort (no crisis or war period, first or second world war, and the great depression), living with biological parents at the age of ten (both parents, mother or father, or without parents). Birth cohort was measured at every wave during follow-up, and living with biological parents was measured as part of the SHARELIFE module in wave 3. As indicators of lifestyle, the following health behaviour and condition variables were included in the analyses: body mass index (BMI; ≤ 24.9, 25.0–29.9, ≥ 30.0 kg/m^2^), smoking, number of chronic conditions, and physical activity (see Online Resource 2 for details).

As potential mediators, the following indicators of ASCs were included: participant’s level of education (low or high), participant’s main occupation (high or low skill), and satisfaction with household income (derived from the question “Is household able to make ends meet?”, ranging from 1, with great difficulty, to 4, easily). Education was based on the highest educational attainment, along the ISCED classification. Participants having reached tertiary education level were classified as “high” and others as “low and middle”. Main occupation—high or low skilled—was based on the International Standard Classification of Occupations (International Labour Office 2012) of the main job during the working life, derived from the questions “Which of the jobs you have told me about was the final job of your main career or occupation? By this we mean the last job in the career or the occupation that took up most of your working life, even though you might have had other jobs afterwards”. We considered occupation as a proxy of skills that individuals can develop over their life course. Participants who reported never having performed paid work were classified as low skill.

### Statistical analyses

Prevalence of cancer was based on proportions of respondents reporting the first occurrence of cancer. Differences in cancer types by CSCs were assessed with Chi-square tests (all cancer types, breast, and prostate) and Fisher’s exact tests (all other types of cancer: colon or rectum, etc.). Duration started with date of birth and ended with age of first cancer, or with end of follow-up or death, whichever came first. For ease of visualization, Kaplan–Meier curves for the cumulative proportion of cancer-free participants were plotted between 50 and 105 years.

The association of CSCs with first self-reported diagnosis of cancer (overall and by site) was assessed separately by sex by using Cox proportional-hazards regression, adjusting for the confounders age, birth cohort, living with biological parents, and reason for attrition (Model 0). Hazard ratios and 95% confidence intervals were estimated. Because the prevalence was low, such analyses by cancer site were limited to the following sites: colon or rectum, skin, breast and cervix (women only), and prostate.

The following models were used to examine whether CSCs remained associated when adjusting for ASCs and health situation: Models 1–3 adjusted for ASCs (M1: education; M2: main occupation; M3: income; M4: adjusted for all three ASCs). A fifth model further included health status and health behaviours (BMI, smoking status, chronic health conditions, and physical activity). We verified the assumptions for Cox models using both visual inspection of residuals and statistical tests and confirmed the validity of the Cox models used in this study. We used strata for country in the Cox regression analysis to allow for varying baseline hazards. To assess the impact of including a retrospective assessment of cancer (for participants who reported a cancer before inclusion in SHARE), we ran two sensitivity analyses: one excluding participants with cancer before age 50 and one excluding all participants reporting cancer before inclusion in SHARE to examine the impact of potential reverse causality. Statistical analyses involved use of the R language version 3.4.1.

## Results

### Participant characteristics

A total of 14,836 (56%) women and 11,595 (44%) men were included in the analyses (mean [SD] age 62.2 [10.3] and 63.0 [9.2] years) (Table 1). For both men and women, participants with middle CSCs were the largest group, followed by disadvantaged, while the most advantaged group was the smallest (Table 2). Participant characteristics by CSCs are presented in Online Resource 3. Among the included participants, 1517 (10.2%) women and 1335 (11.5%) men reported having or having had a first diagnosis of cancer (Table 2). By cancer site, the numbers for colon or rectal cancer were 142 women and 151 men; skin cancer, 119 women and 113 men; breast cancer, 618 women; cervical cancer, 123 women, and prostate cancer, 368 men. Among women, the distribution of overall cancer, breast, colon or rectum, skin, and cervix cancer was different by CSCs strata (Table 2). Among men, no difference was observed.

**Table 1:** Participant characteristics, stratified by gender, from the Survey of Health, Ageing and Retirement in Europe (collected in Austria, Belgium, Czech Republic, Denmark, France, Germany, Greece, Ireland, Italy, the Netherlands, Poland, Spain, Sweden, and Switzerland, 2016)

	Women (*n* = 14,836)	Men (*n* = 11,595)
*Confounders*		
Age at baseline, years (SD)	62.2 (10.3)	63.0 (9.2)
Birth cohort		
No war and no great depression	7018 (51.6)	5499 (49.0)
War	3335 (24.5)	2852 (25.4)
Great depression	3236 (23.8)	2861 (25.5)
Living with biological parents		
Both parents	13,413 (90.4)	10,504 (90.6)
One biological parent	1127 (7.6)	881 (7.6)
Without biological parent	296 (2.0)	209 (1.8)
Attrition		
No dropout	10,391 (70.0)	7788 (67.2)
Dropout	3226 (21.7)	2487 (21.4)
Death	1219 (8.2)	1320 (11.4)
*Covariates*		
BMI, kg/m^2^		
≤ 24.9	6129 (41.9)	3492 (30.5)
25.0–29.9	5701 (39.0)	5937 (51.8)
≥ 30.0	2794 (19.1)	2024 (17.7)
Smoking status at baseline		
Never smoker	5892 (66.7)	2434 (35.7)
Ex-smoker	1521 (17.2)	2800 (41.0)
Current smoker	1420 (16.1)	1587 (23.3)
No. of chronic conditions		
< 2	8196 (55.3)	7165 (61.8)
≥ 2	6629 (44.7)	4422 (38.2)
Physical activity		
Low	9989 (67.4)	8404 (72.5)
High	4825 (32.6)	3181 (27.5)
*Adult socioeconomic status*		
Level of education		
Low	11,849 (83.0)	8532 (76.8)
High	2428 (17.0)	2571 (23.2)
Main occupation class		
Low skill	12,291 (83.7)	7883 (69.1)
High skill	2395 (16.3)	3523 (30.9)
Household income (able to make ends meet)		
Easily	5175 (34.9)	4503 (38.9)
Fairly easily	4529 (30.6)	3603 (31.1)
With some difficulty	3369 (22.8)	2396 (20.7)
With great difficulty	1734 (11.7)	1082 (9.3)

Data are *n* (%) unless indicated

*BMI* body mass index, *SD* standard deviation

**Table 2:** Cancer incidence overall and by site, stratified by gender and childhood socioeconomic conditions (the Survey of Health, Ageing and Retirement in Europe, collected in Austria, Belgium, Czech Republic, Denmark, France, Germany, Greece, Ireland, Italy, the Netherlands, Poland, Spain, Sweden, and Switzerland, 2016)

	Women (*n* = 14,836)						
	All	Most advantaged (*n* = 821)	Advantaged (*n* = 2674)	Middle (*n* = 4798)	Disadvantaged (*n* = 3771)	Most disadvantaged (*n* = 2772)	*p*
All cancer types	1517 (10.2)	92 (11.2)	338 (12.6)	454 (9.5)	363 (9.6)	270 (9.7)	< 0.001
Breast	618 (4.2)	49 (6.0)	142 (5.3)	183 (3.8)	150 (4.0)	94 (3.4)	< 0.001
Prostate	–	–	–	–	–	–	
Colon or rectum	142 (1.0)	6 (0.7)	39 (1.5)	42 (0.9)	27 (0.7)	28 (1.0)	0.04
Skin	119 (0.8)	7 (0.9)	37 (1.4)	40 (0.8)	26 (0.7)	9 (0.3)	< 0.001
Lung	35 (0.2)	1 (0.1)	8 (0.3)	9 (0.2)	9 (0.2)	8 (0.3)	0.82
Cervix	123 (0.8)	6 (0.7)	38 (1.4)	26 (0.5)	32 (0.8)	21 (0.8)	0.004
Kidney	31 (0.2)	2 (0.2)	6 (0.2)	8 (0.2)	13 (0.3)	2 (0.1)	0.15
Stomach	30 (0.2)	0 (0.0)	8 (0.3)	7 (0.1)	10 (0.3)	5 (0.2)	0.38
Ovary	102 (0.7)	5 (0.6)	22 (0.8)	26 (0.5)	30 (0.8)	19 (0.7)	0.56
Leukaemia	23 (0.2)	0 (0.0)	4 (0.1)	5 (0.1)	8 (0.2)	6 (0.2)	0.52
Endometrium	77 (0.5)	3 (0.4)	18 (0.7)	23 (0.5)	19 (0.5)	14 (0.5)	0.81
Bladder	25 (0.2)	1 (0.1)	6 (0.2)	6 (0.1)	10 (0.3)	2 (0.1)	0.31
Thyroid	44 (0.3)	1 (0.1)	8 (0.3)	14 (0.3)	13 (0.3)	8 (0.3)	0.94
Liver	23 (0.2)	1 (0.1)	6 (0.2)	5 (0.1)	9 (0.2)	2 (0.1)	0.32

	Men (*n* = 11,595)						
	All	Most advantaged (*n* = 654)	Advantaged (*n* = 2123)	Middle (*n* = 3610)	Disadvantaged (*n* = 2918)	Most disadvantaged (*n* = 2290)	*p*
All cancer types	1335 (11.5)	77 (11.7)	254 (12.0)	390 (10.8)	327 (11.2)	287 (12.5)	0.30
Breast	16 (0.1)	2 (0.3)	4 (0.2)	7 (0.2)	2 (0.1)	1 (0.0)	0.28
Prostate	368 (3.2)	23 (3.5)	84 (4.0)	117 (3.2)	85 (2.9)	59 (2.6)	0.10
Colon or rectum	151 (1.3)	9 (1.4)	36 (1.7)	38 (1.1)	31 (1.1)	37 (1.6)	0.11
Skin	113 (1.0)	10 (1.5)	27 (1.3)	37 (1.0)	24 (0.8)	15 (0.7)	0.11
Lung	82 (0.7)	5 (0.8)	12 (0.6)	25 (0.7)	23 (0.8)	17 (0.7)	0.90
Cervix	–	–	–	–	–	–	
Kidney	48 (0.4)	4 (0.6)	9 (0.4)	16 (0.4)	9 (0.3)	10 (0.4)	0.76
Stomach	43 (0.4)	3 (0.5)	7 (0.3)	14 (0.4)	8 (0.3)	11 (0.5)	0.74
Ovary	–	–	–	–	–	–	
Leukaemia	41 (0.4)	2 (0.3)	11 (0.5)	8 (0.2)	16 (0.5)	4 (0.2)	0.06
Endometrium	–	–	–	–	–	–	
Bladder	67 (0.6)	6 (0.9)	13 (0.6)	21 (0.6)	15 (0.5)	12 (0.5)	0.76
Thyroid	6 (0.1)	0 (0.0)	1 (0.0)	2 (0.1)	2 (0.1)	1 (0.0)	1.00
Liver	31 (0.3)	2 (0.3)	6 (0.3)	12 (0.3)	5 (0.2)	6 (0.3)	0.77

Data are *n* (%); *p* values come from Chi-square tests for all cancer types, breast, and prostate cancers, and from Fisher’s exact tests for other cancer types (colon or rectum, etc.)

### Association of life course socioeconomic conditions on overall cancer

**Fig. 1:**
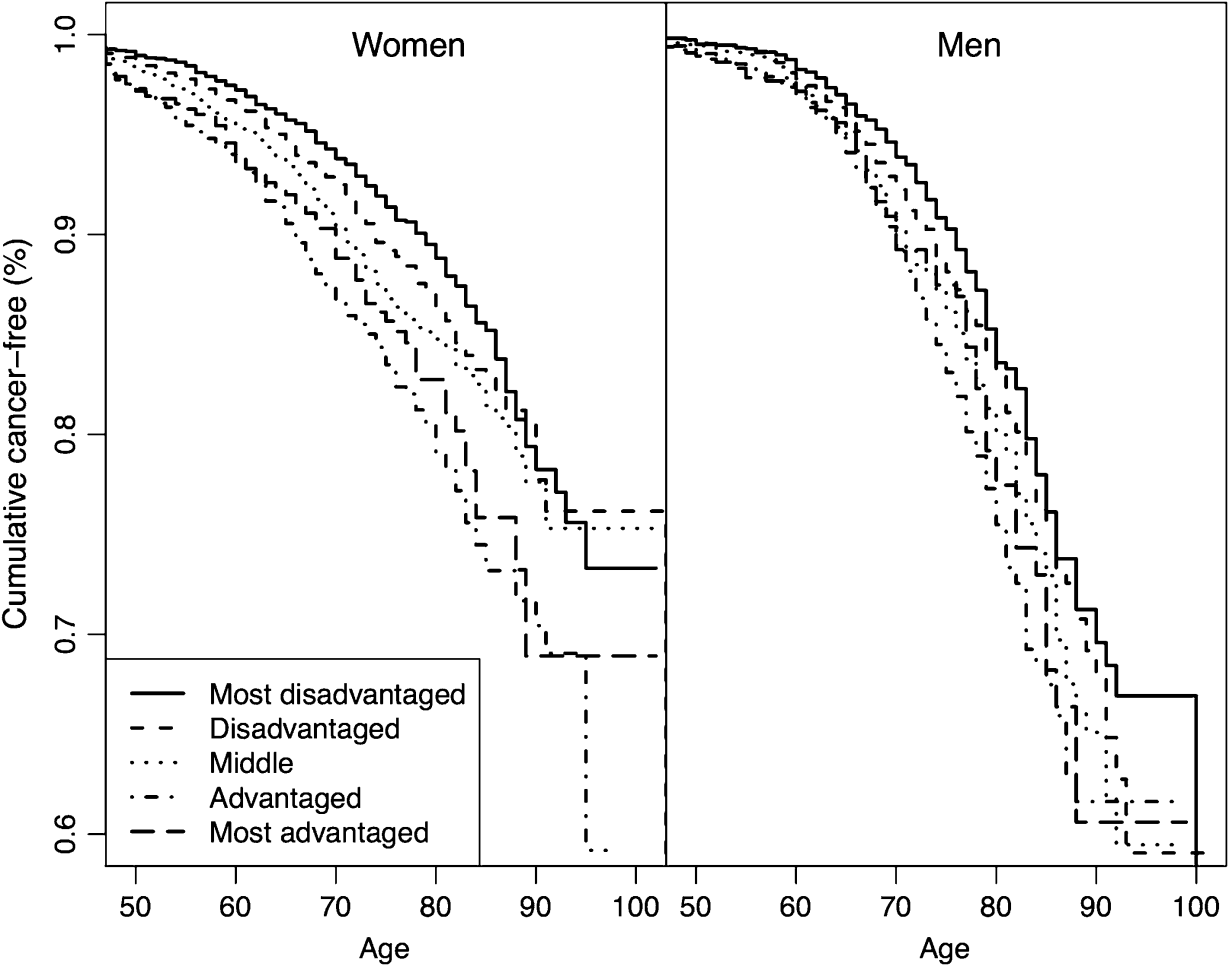
Kaplan–Meier curve for the cumulative proportion of cancer-free participants over time by gender and childhood socioeconomic conditions (the Survey of Health, Ageing and Retirement in Europe, collected in Austria, Belgium, Czech Republic, Denmark, France, Germany, Greece, Ireland, Italy, the Netherlands, Poland, Spain, Sweden, and Switzerland, 2016). *Note*: in the analyses, age started at birth, but is presented from age 50 onwards in the figure

Figure 1 shows the Kaplan–Meier curve for the cumulative proportion of cancer-free participants between 50 and 105 years by sex and CSCs. Both men and women with the most disadvantaged CSCs were most likely to be cancer-free. Differences in CSCs seemed to slightly disappear over time; however, for people ≥ 80 years old, women with advantaged CSCs seemed most likely to have had cancer. For people < 75 years old, men with advantaged CSCs seemed most likely to have had cancer. For men ≥ 75 years old, those from middle and most advantaged CSCs were most likely to have had a cancer. In women, like in men, differences in CSCs slightly disappear in very old age, except that men with advantaged CSCs who were ≥ 90 years old were most likely to still be cancer-free.

The results of multivariable analyses are given in Table 3. When adjusting for confounders and attrition, as compared with women with the most disadvantaged CSCs, those with more advantaged CSCs were more likely to have had cancer, especially those with advantaged CSCs (HR 1.50, 1.72, and 1.18, for most advantaged, advantaged, and middle CSCs, respectively, Table 3, Model 0). When adjusting for ASCs, sociodemographic characteristics, health status, and health behaviours, the effects remained significant (HR 1.49, 1.70, and 1.19, for the most advantaged, advantaged, and middle CSCs, respectively, Table 3, Model 5). For men, results were similar. Men with more advantaged CSCs were more likely to have had a cancer as compared with men with the most disadvantaged CSCs. Those with advantaged CSCs were most likely to have had a cancer (HR 1.48, Table 3, Model 0, and 1.36, Table 3, Model 5). ASCs did not change the effect of CSCs on cancer onset later in life.

**Table 3:** Associations between childhood socioeconomic conditions (CSCs) and cancer overall and by site at older age, stratified by gender (the Survey of Health, Ageing and Retirement in Europe, collected in Austria, Belgium, Czech Republic, Denmark, France, Germany, Greece, Ireland, Italy, the Netherlands, Poland, Spain, Sweden, and Switzerland, 2016)

Model/CSCs	Women			Men		
	All cancer types	Colon or rectum	Skin	All cancer types	Colon or rectum	Skin
	(*n* = 1517)	(*n* = 142)	(*n* = 119)	(*n* = 1335)	(*n* = 151)	(*n* = 113)
Model 0						
Most advantaged	1.50 (1.18–191)	0.61 (0.25–1.48)	2.28 (0.84–6.17)	1.34 (1.04–1.73)	0.61 (0.29–1.27)	1.99 (0.89–4.45)
Advantaged	1.72 (1.46–2.04)	1.14 (0.69–1.87)	3.10 (1.48–6.48)	1.48 (1.24–1.76)	0.80 (0.50–1.27)	1.73 (0.91–3.29)
Middle	1.18 (1.01–1.38)	0.91 (0.56–1.49)	2.56 (1.23–5.33)	1.25 (1.07–1.46)	0.60 (0.38–0.96)	1.70 (0.92–3.12)
Disadvantaged	1.09 (0.93–1.28)	0.66 (0.39–1.14)	1.99 (0.93–4.27)	1.11 (0.95–1.31)	0.62 (0.38–1.00)	1.30 (0.68–2.50)
Most disadvantaged	1	1	1	1	1	1
Model 1						
Most advantaged	1.44 (1.12–1.87)	0.59 (0.24–1.46)	2.30 (0.84–6.35)	1.26 (0.97–1.65)	0.52 (0.24–1.13)	1.52 (0.64–3.62)
Advantaged	1.70 (1.42–2.02)	1.07 (0.63–1.80)	3.13 (1.48–6.59)	1.43 (1.19–1.71)	0.71 (0.42–1.17)	1.56 (0.80–3.03)
Middle	1.19 (1.01–1.39)	0.89 (0.54–1.47)	2.55 (1.22–5.33)	1.20 (1.02–1.42)	0.59 (0.37–0.95)	1.67 (0.91–3.09)
Disadvantaged	1.11 (0.94–1.31)	0.67 (0.39–1.15)	1.95 (0.91–4.18)	1.11 (0.94–1.30)	0.63 (0.39–1.03)	1.33 (0.69–2.55)
Most disadvantaged	1	1	1	1	1	1
Model 2						
Most advantaged	1.44 (1.12–1.85)	0.58 (0.23–1.45)	1.91 (0.69–5.30)	1.20 (0.92–1.56)	0.53 (0.25–1.14)	2.06 (0.88–4.82)
Advantaged	1.67 (1.41–1.99)	1.12 (0.67–1.88)	2.82 (1.33–5.96)	1.36 (1.13–1.63)	0.70 (0.42–1.16)	1.81 (0.92–3.56)
Middle	1.16 (0.99–1.37)	0.91 (0.55–1.50)	2.40 (1.15–5.03)	1.18 (1.00–1.39)	0.57 (0.35–0.91)	1.77 (0.94–3.31)
Disadvantaged	1.10 (0.93–1.29)	0.66 (0.38–1.14)	1.90 (0.89–4.07)	1.09 (0.92–1.28)	0.60 (0.37–0.98)	1.37 (0.70–2.65)
Most disadvantaged	1	1	1	1	1	1
Model 3						
Most advantaged	1.43 (1.11–1.83)	0.58 (0.24–1.44)	1.88 (0.68–5.16)	1.28 (0.99–1.66)	0.58 (0.27–1.25)	1.90 (0.83–4.37))
Advantaged	1.65 (1.39–1.97)	1.10 (0.66–1.86)	2.66 (1.26–5.64)	1.42 (1.19–1.70)	0.76 (0.46–1.25)	1.64 (0.84–3.20)
Middle	1.14 (0.97–1.34)	0.89 (0.53–1.48)	2.24 (1.06–4.72)	1.21 (1.03–1.42)	0.59 (0.36–0.95)	1.64 (0.88–3.06)
Disadvantaged	1.07 (0.91–1.26)	0.66 (0.38–1.15)	1.85 (0.86–3.99)	1.10 (0.93–1.29)	0.61 (0.37–0.99)	1.23 (0.63–2.38)
Most disadvantaged	1	1	1	1	1	1
Model 4						
Most advantaged	1.38 (1.06–1.80)	0.58 (0.23–1.48)	1.71 (0.61–4.80)	1.15 (0.87–1.52)	0.48 (0.22–1.08)	1.62 (0.66–4.00)
Advantaged	1.63 (1.36–1.96)	1.07 (0.62–1.86)	2.55 (1.19–5.46)	1.31 (1.08–1.59)	0.65 (0.38–1.12)	1.64 (0.81–3.31)
Middle	1.15 (0.97–1.36)	0.90 (0.53–1.52)	2.13 (1.01–4.50)	1.13 (0.95–1.34)	0.57 (0.35–0.93)	1.74 (0.92–3.31)
Disadvantaged	1.10 (0.93–1.30)	0.68 (0.39–1.19)	1.73 (0.80–3.72)	1.07 (0.91–1.27)	0.63 (0.38–1.03)	1.33 (0.68–2.62)
Most disadvantaged	1	1	1	1	1	1
Model 5						
Most advantaged	1.25 (0.91–1.72)	0.54 (0.18–1.65)	2.60 (0.78–8.72)	1.13 (0.80–1.59)	0.41 (0.15–1.11)	1.50 (0.51–4.36)
Advantaged	1.50 (1.19–1.88)	0.83 (0.41–1.72)	3.55 (1.41–8.93)	1.37 (1.08–1.74)	0.61 (0.31–1.18)	1.41 (0.60–3.32)
Middle	0.94 (0.76–1.16)	0.86 (0.44–1.69)	2.55 (0.99–6.58)	1.18 (0.95–1.46)	0.49 (0.27–0.91)	1.32 (0.59–2.93)
Disadvantaged	1.14 (0.93–1.39)	0.69 (0.35–1.37)	1.74 (0.68–4.46)	1.09 (0.88–1.35)	0.51 (0.27–0.95)	1.49 (0.66–3.37)
Most disadvantaged	1	1	1	1	1	1

Data are hazard ratios (HRs) and 95% confidence intervals (CIs), 1 reference category

*Model 0* adjusted for confounders and attrition: age, birth cohort, living with biological parents, reason for dropout if drop out, *Model 1* M0 + adjusted for education, *Model 2* M1 + adjusted for main occupational class, *Model 3* M2 + adjusted for household income, *Model 4* M3 + adjusted for all life course socioeconomic conditions, *Model 5* M4 + adjusted for sociodemographics, health status, health behaviours

### Association of life course socioeconomic conditions on cancer by site

Table 3 reports results from multivariable Cox proportional-hazard regression analyses for non-sex-specific cancers, and Table 4 reports these results for sex-specific cancers. Women with advantaged and middle CSCs were more likely to have had skin cancer (HR 3.10 and 2.56, respectively, Table 3, Model 0). This association remained significant when controlling for ASCs (HR 2.55 and 2.13, respectively, Table 3, Model 4). Risk of breast cancer was 1.53 times more likely for women with the most advantaged than most disadvantaged CSCs (Table 4, Model 0). This remained significant after adjusting for ASCs (HR 1.49, Table 4, Model 4). Additionally, we found no significant associations for colon and rectal or cervical cancer.

**Table 4:** Associations between CSCs and gender-specific cancer at older age (the Survey of Health, Ageing and Retirement in Europe, collected in Austria, Belgium, Czech Republic, Denmark, France, Germany, Greece, Ireland, Italy, the Netherlands, Poland, Spain, Sweden, and Switzerland, 2016)

Model/CSCs	Breast^a^	Cervix^a^	Prostate^b^
	(*n* = 618)	(*n* = 123)	(*n* = 368)
Model 0			
Most advantaged	1.53 (1.07–2.19)	0.77 (0.30–1.96)	1.18 (0.72–1.91)
Advantaged	0.99 (0.75–1.30)	1.33 (0.74–2.41)	1.29 (0.92–1.81)
Middle	1.01 (0.78–1.31)	0.75 (0.40–1.40)	1.31 (0.95–1.80)
Disadvantaged	1.05 (0.81–1.37)	1.14 (0.63–2.08)	1.12 (0.80–1.56)
Most disadvantaged	1	1	1
Model 1			
Most advantaged	1.48 (1.01–2.15)	0.67 (0.26–1.76)	1.13 (0.68–1.88)
Advantaged	0.98 (0.74–1.30)	1.22 (0.66–2.25)	1.22 (0.86–1.74)
Middle	1.00 (0.77–1.30)	0.71 (0.38–1.33)	1.21 (0.88–1.68)
Disadvantaged	1.06 (0.81–1.39)	1.10 (0.61–2.01)	1.11 (0.79–1.55)
Most disadvantaged	1	1	1
Model 2			
Most advantaged	1.61 (1.11–2.32)	0.85 (0.32–2.26)	1.11 (0.67–1.84)
Advantaged	1.00 (0.76–1.32)	1.49 (0.79–2.79)	1.22 (0.86–1.74)
Middle	0.99 (0.76–1.28)	0.84 (0.44–1.62)	1.28 (0.93–1.77)
Disadvantaged	1.02 (0.78–1.33)	1.27 (0.68–2.36)	1.10 (0.79–1.55)
Most disadvantaged	1	1	1
Model 3			
Most advantaged	1.48 (1.02–2.14)	0.86 (0.33–2.26)	1.14 (0.69–1.89)
Advantaged	0.97 (0.73–1.28)	1.47 (0.79–2.69)	1.26 (0.89–1.80)
Middle	0.99 (0.76–1.30)	0.82 (0.43–1.55)	1.29 (0.93–1.79)
Disadvantaged	1.04 (0.79–1.36)	1.18 (0.65–2.17)	1.11 (0.79–1.56)
Most disadvantaged	1	1	1
Model 4			
Most advantaged	1.49 (1.01–2.19)	0.83 (0.30–2.28)	1.09 (0.64–1.84)
Advantaged	0.97 (0.72–1.30)	1.48 (0.77–2.85)	1.16 (0.80–1.68)
Middle	0.96 (0.73–1.26)	0.86 (0.44–1.68)	1.19 (0.85–1.66)
Disadvantaged	1.02 (0.78–1.33)	1.27 (0.68–2.38)	1.10 (0.78–1.55)
Most disadvantaged	1	1	1
Model 5			
Most advantaged	1.28 (0.79–2.08)	0.76 (0.22–2.69)	1.37 (0.74–2.56)
Advantaged	0.95 (0.67–1.35)	1.45 (0.65–3.26)	1.16 (0.73–1.83)
Middle	0.87 (0.62–1.23)	0.70 (0.29–1.72)	1.40 (0.92–2.13)
Disadvantaged	0.96 (0.69–1.32)	1.19 (0.55–2.60)	1.27 (0.83–1.93)
Most disadvantaged	1	1	1

Data are hazard ratios (HRs) and 95% confidence intervals (CIs), 1 reference category

*Model 0* adjusted for confounders and attrition: age, birth cohort, living with biological parents, reason for dropout if drop out, *Model 1* M0+ adjusted for education, *Model 2* M1+ adjusted for main occupational class, *Model 3* M2+ adjusted for household income, *Model 4* M3+ adjusted for all life course socioeconomic conditions, *Model 5* M4+ adjusted for sociodemographics, health status, health behaviours

^a^Only in women

^b^Only in men

For men, results for colon and rectal cancer were opposite from those for cancer overall. Men with middle CSCs were less likely to have had colon or rectal cancer than those with the most advantaged CSCs, and ASCs did not change this effect (HR 0.57, Table 3, Model 4). We found no significant associations for skin and prostate cancer.

### Sensitivity analysis

None of the tests for violation of the proportional-hazards assumption gave significant results. Results from the Cox models including strata for country showed a similar pattern of results, with HRs increasing with CSCs up to advantaged and a slight decrease for the most advantaged. However, the HRs were closer to the null and that for the advantaged group were often the only one significantly different from 1. The two sensitivity analyses showed similar results with the same gradient, though closer to the null.

## Discussion

In this study, we examined whether CSCs were associated with cancer onset in later life by using longitudinal data for older adults, ≥ 50 years old, from 14 countries across Europe. The second aim was to test whether this effect remained after adjusting for adult life conditions, ASCs, for cancer overall and by site. Overall, both men and women with the most disadvantaged CSCs were most likely to be cancer-free over time, but results vary by cancer sites and by sex. Women with advantaged and middle CSCs were more than twice as likely to have had skin cancer than those with the most disadvantaged CSCs. As compared with men or women with the most disadvantaged CSCs, men with middle CSCs were half as likely to have had colon or rectal cancer and women with the most advantaged CSCs were more likely to have had breast cancer. Our findings suggested no mediating effects of ASCs.

Studies on socioeconomic inequalities in overall cancer mortality and survival show inconsistent results, as site-specific cancers have different aetiologies and mortality and survival are related to incidence and factors that influence survival such as health care (Vohra et al. 2016). However, findings of two other studies also found lower risk of overall cancer in people from more advantaged conditions (Lawlor et al. 2006; Strand and Kunst 2007). For site-specific cancers, most evidence from previous studies are inconsistent and imprecise (Vohra et al. 2016). Three studies on risk of bowel and rectal cancer support our findings that poorer CSCs are associated with higher risk (de Kok et al. 2008; Naess et al. 2004, 2007). For skin and breast cancer, previous studies on cancer incidence are in line with our findings that can partly be explained by socioeconomic-related risk factors, such as higher exposure to ultraviolet radiation for skin, and older age at first birth for breast cancer (Bryere et al. 2016; de Kok et al. 2008; Pudrovska and Anikputa 2012). Bryere and colleagues also found an association between low social class and higher risk of cervical and lower risk of prostate cancer (Bryere et al. 2016). Our findings do not show this, which may be due to low number of cases by cancer subtype and CSCs. Like de Kok and colleagues, we found no mediation by ASCs (de Kok et al. 2008). The results support studying cancer from a life course perspective to find possible pathways by different cancer-specific risk factors and exposure by social class over the life course.

A possible explanation for the findings might be socioeconomic differences in health behaviours, such as cancer screening, and cancer risk factors. Some studies found that individuals from low socioeconomic status may have barriers that impact participation in screening and thus detection (Deding et al. 2017; Wang et al. 2017). This situation may lead to higher cancer rates for individuals from high than low socioeconomic status that are actually caused by increased detection and not true differences. Regarding risk factors, women from higher socioeconomic class show increased alcohol intake and age at first offspring birth and reduced parity, which are related to increased risk of breast cancer (Lundqvist et al. 2016). Similarly, the incidence of skin cancer with high socioeconomic class may be explained by holidays abroad and exposure to UV irradiation (Shack et al. 2008).

A strength of this study was the use of a European longitudinal database with rich information on life course socioeconomic conditions and a potential observation period of 12 years. The sample size of this database may be sufficient to draw convincing conclusions for overall cancer. To limit the risk of misclassification bias, we used pre-defined and previously used methods for defining and analysing socioeconomic conditions. Additionally, we tried to minimize health selection bias by including respondents who participated in only one wave and completed the retrospective life course module. Finally, our sample includes participants from 14 European countries. This has the advantages to capture a more representative sample of the general population and to increase statistical power and at the same time the disadvantage to increase variability due to the heterogeneity in terms of cancer country profile within Europe (Ferlay et al. 2013).

This study has three main limitations. First, the self-reported cancer diagnosis instead of cancer registry data, which may imply reporting bias. Previous studies found an overall rate of false-negative self-reporting of 39.2%, with a wide variation by cancer site (Desai et al. 2001). Older age may also be associated with more frequent false-positive reporting (Loh et al. 2014). However, studies showed that respondents can accurately report a past cancer diagnosis, especially for breast, prostate, and colon cancer, with an overall sensitivity of self-reported cancer of up to 89% (Bergmann et al. 1998; Loh et al. 2014). Second, by its design (inclusion of respondents aged 50 years old and older at baseline), SHARE is impeded by a cancer survivor bias. The importance of this bias may affect our finding, especially among the oldest old. Nevertheless, this bias is also limited as (1) the probability of dying from cancer before age 50 year is low, and (2) the overall cancer death rate in Europe and USA is decreasing (Malvezzi et al. 2015; Siegel et al. 2018). Third, the life course span of the three ASCs raises the question of causality of exposure, mediators, and the outcome (e.g. onset of cancer before educational achievement) and of potential reverse causality. Twenty-three participants reported cancer before the age of 30 and 44 before the age of 50, so it is reasonable to assume that reverse causality on education and main occupation will not bias the results. Concerning satisfaction with income (a time-varying mediator measured at each wave, i.e. at age 50 and later), it is reasonable to think that a part of the income and cancer onset association is influenced by reverse association (i.e. cancer causes income to decrease). We preferred keeping this important measure of ASCs in the analyses. This temporality issue should reinforce the effect size of income because the reverse causation association is probably stronger than the direct causation (i.e. income causes cancer). If the association of income and cancer is overestimated, then the mediating effect of income on the association of CSCs and cancer should also be overestimated. Thus, still finding an association between CSCs and cancer after adjusting for income is a strong sign of the independent impact of CSCs on later health.

Additionally, we did not have information on all cancer risk factors and confounders, such as genes, perinatal, and environmental factors, such as air pollution, pesticides, and herbicides (IARC Publications 2013). Another limitation is the retrospective and self-reported information on CSCs and ASCs, which may be subject to recall bias. Still, previous studies found evidence for the accuracy of recall of simple measures of socioeconomic conditions in a survey of older adults (Lacey et al. 2012). Also, sample sizes for site-specific cancers stratified by CSCs differ by cancer type and might have insufficient power to detect an association. Finally, given the longitudinal data, participants dropped out or died during follow-up, which may influence the results. For example, Bouchardy et al. (2006) reported an increased risk of dying from breast cancer for patients with low versus high social class. To limit this bias, we adjusted for attrition in the analyses. By including attrition in all models, we adjusted for mediator-outcome confounding, although this statistical adjustment did not solve the issue of missing data due to attrition.

### Conclusions

The present study is the first longitudinal European study to analyse the direct association of CSCs with cancer later in life as well as via pathways exploring the role of ASCs as a mediator. Our results suggest an association of advantaged CSCs with an increased overall cancer onset in older age, but results vary by cancer sites and by sex. Additionally, pathways to cancer may start in early life and ASCs does not completely mediate this relation.

The findings give evidence of the long-term impact of CSCs as well as the influence of ASCs on adult health. The findings may in turn help in developing interventions and targeting groups at an increased risk of not participating in cancer screening programmes and/or developing cancer. Future studies are warranted to examine the relation of CSCs with cancers by site in a larger sample to increase power and with detailed information on health behaviours and risk exposure to explore more pathways by including a formal test of mediation. More evidence on the association between CSCs and the risk of specific cancers could help better identify and understand the relation, which in turn could lead to the improvement and tailoring of prevention programmes.

## Electronic supplementary material

Below is the link to the electronic supplementary material.

Supplementary material 1 (DOCX 35 kb)
