# Supplementary material for: Effect of childhood socioeconomic conditions on cancer onset in later life: an ambidirectional cohort study
Source: Int J Public Health. 2018 May 17;63(7):799–810. doi: 10.1007/s00038-018-1111-9 (PMC6154039; doi:10.1007/s00038-018-1111-9)
Supplement: Supplementary file 1 — Supplementary material 1 (DOCX 35 kb) [file 38_2018_1111_MOESM1_ESM.docx]

**Article title:** Effect of childhood socioeconomic conditions on cancer onset in later life: an ambidirectional cohort study

**Journal name:** International Journal of Public Health

**Author names:** Bernadette W.A. van der Linden, Delphine S. Courvoisier, Boris Cheval, Stefan Sieber, Piet Bracke, Idris Guessous, Claudine Burton-Jeangros, Matthias Kliegel, Stéphane Cullati

**Affiliation of corresponding author:** Swiss NCCR “LIVES - Overcoming Vulnerability: Life Course Perspectives”, University of Geneva, Switzerland; Center for the Interdisciplinary Study of Gerontology and Vulnerability, University of Geneva, Geneva, Switzerland

**E-mail address of corresponding author:** rose.vanderlinden@unige.ch

**Online Resource 1** Operationalization of childhood socioeconomic conditions

The occupational position of the main breadwinner was constructed based on a reclassification of the 10 main occupational groups of the International Standard Classification of Occupations (ISCO-08) according to their skill levels (1, 2). The first and second skill levels were classified as “low” and the third and fourth skill levels as “high” occupational position. For the number of books at home, a binary item was constructed with the category 0–10 books used as indicator of social disadvantage (3). To construct a measure of overcrowding, we combined information on the number of people living in the household and the number of rooms in the house (excluding the kitchen, bathrooms, and hallways), with more than one person per room considered overcrowding (4). Housing quality was assessed by a fixed bath, cold and hot running water supply, indoor toilet, and central heating. If none of these were present, the household was considered disadvantaged (5). By combining the four indicators, a categorical variable for CSCs was computed, with the categories most advantaged (no disadvantaged socioeconomic conditions at age 10), advantaged (1 of the indicators), middle (2 indicators), disadvantaged (3 indicators), and most disadvantaged (all 4 indicators of disadvantaged socioeconomic conditions).

**References**

1. Wahrendorf M, Blane D, Bartley M, Dragano N, Siegrist J. Working conditions in mid-life and mental health in older ages. Advances in life course research. 2013;18(1):16-25.

2. International Labour Office. International Standard Classification of Occupations: ISCO-08. Geneva: ILO: International Labour Organization,; 2012.

3. Evans MDR, Kelley J, Sikora J, Treiman DJ. Family scholarly culture and educational success: Books and schooling in 27 nations. Research in Social Stratification and Mobility. 2010;28(2):171-97.

4. Marsh A, Gordon, D., Pantazis, C., Heslop, P. Home Sweet Home? The impact of poor housing on health. Bristol: The Policy Press, University of Bristol; 1999.

5. Dedman DJ, Gunnell D, Davey Smith G, Frankel S. Childhood housing conditions and later mortality in the Boyd Orr cohort. Journal of epidemiology and community health. 2001;55(1):10-5.

**Article title:** Effect of childhood socioeconomic conditions on cancer onset in later life: an ambidirectional cohort study

**Journal name:** International Journal of Public Health

**Author names:** Bernadette W.A. van der Linden, Delphine S. Courvoisier, Boris Cheval, Stefan Sieber, Piet Bracke, Idris Guessous, Claudine Burton-Jeangros, Matthias Kliegel, Stéphane Cullati

**Affiliation of corresponding author:** Swiss NCCR “LIVES - Overcoming Vulnerability: Life Course Perspectives”, University of Geneva, Switzerland; Center for the Interdisciplinary Study of Gerontology and Vulnerability, University of Geneva, Geneva, Switzerland

**E-mail address of corresponding author:** [rose.vanderlinden@unige.ch](mailto:rose.vanderlinden@unige.ch)

**Online Resource 2** Operationalization covariates and confounders

Birth cohort was categorized into; no crisis or war period [i.e., born before 1914, between 1919 and 1928, or after 1945], first or second world war [i.e. born between 1914 and 1918 or between 1939 and 1945], and the Great Depression [i.e., born between 1929 and 1938]. As indicators of lifestyle, the following health behavior and condition variables were included in the analyses; body mass index (BMI; ≤24.9, 25.0-29.9, ≥30.0 kg/m^2^), measured in every wave except in wave 3. The average over all waves was calculated in order to obtain a time unvarying variable and to not lose observations. Further, smoking, number of chronic conditions, and physical activity (low, high) were added. Smoking status at baseline assessed if respondents were never, ex- or current smoker. For the average number of chronic conditions, a score was computed based on the following conditions across all waves: stroke, heart attack, hypertension, diabetes, Parkinson disease, and asthma. Physical activity was based on two items assessing the level of daily life physical activity in every wave except wave 3. Vigorous physical activity was assessed with the question “How often do you engage in vigorous physical activity, such as sports, heavy housework, or a job that involves physical labour?” Moderate physical activity was assessed with the question “How often do you engage in activities that require a low or moderate level of energy, such as gardening, cleaning the car, or walking?”. Answers were based on a 4-point scale (1, more than once a week; 2, once a week; 3, one to three times a month; 4, hardly ever, or never). Participants were classified as having low physical activity if they did not engage in any activity more than once a week. Average scores over the participant’s follow-up period were obtained to measure their physical activity.

**Article title:** Effect of childhood socioeconomic conditions on cancer onset in later life: an ambidirectional cohort study

**Journal name:** International Journal of Public Health

**Author names:** Bernadette W.A. van der Linden, Delphine S. Courvoisier, Boris Cheval, Stefan Sieber, Piet Bracke, Idris Guessous, Claudine Burton-Jeangros, Matthias Kliegel, Stéphane Cullati

**Affiliation of corresponding author:** Swiss NCCR “LIVES - Overcoming Vulnerability: Life Course Perspectives”, University of Geneva, Switzerland; Center for the Interdisciplinary Study of Gerontology and Vulnerability, University of Geneva, Geneva, Switzerland

**E-mail address of corresponding author:** rose.vanderlinden@unige.ch

**Online Resource 3** Participant characteristics by gender and childhood socioeconomic conditions (The Survey of Health, Ageing and Retirement in Europe, collected in Austria, Belgium, Czech Republic, Denmark, France, Germany, Greece, Ireland, Italy, the Netherlands, Poland, Spain, Sweden, and Switzerland, 2016)

|  | Women (n=14825) | | | | | | Men (n=11587) | | | | | |
| --- | --- | --- | --- | --- | --- | --- | --- | --- | --- | --- | --- | --- |
|  | All | Most  advantaged  (n=820) | Advantaged  (n=2672) | Middle  (n=4794) | Disadvantaged  (n=3768) | Most  disadvantaged  (n=2771) | All | Most  advantaged  (n=654) | Advantaged  (n=2121) | Middle  (n=3609) | Disadvantaged  (n=2914) | Most  disadvantaged  (n=2289) |
|  |  |  |  |  |  |  |  |  |  |  |  |  |
| *Confounder***s** |  |  |  |  |  |  |  |  |  |  |  |  |
| Age, years (SD) | 62.2 (10.3) | 59.9 (10.1) | 59.9 (9.7) | 60.6 (9.8) | 63.4 (10.3) | 66.4 (10.2) | 63.0 (9.2) | 61.9 (9.1) | 60.8 (8.7) | 61.5 (8.8) | 63.7 (9.2) | 66.8 (9.1) |
| Birth cohort |  |  |  |  |  |  |  |  |  |  |  |  |
| No war and no  great depression | 7016 (51.7) | 412 (57.8) | 1365 (57.3) | 2404 (55.6) | 1697 (48.2) | 1138 (43.0) | 5497 (49.1) | 336 (53.3) | 1143 (56.5) | 1825 (52.8) | 1318 (46.5) | 875 (38.7) |
| War | 3330 (24.5) | 175 (24.5) | 598 (25.1) | 1059 (24.5) | 882 (25.1) | 616 (23.3) | 2850 (25.4) | 171 (27.1) | 512 (25.3) | 877 (25.4) | 738 (26.1) | 552 (24.4) |
| Great depression | 3234 (23.8) | 126 (17.7) | 418 (17.6) | 858 (19.9) | 939 (26.7) | 893 (33.7) | 2857 (25.5) | 123 (19.5) | 368 (18.2) | 755 (21.8) | 776 (27.4) | 835 (36.9) |
| Living with biological  parents |  |  |  |  |  |  |  |  |  |  |  |  |
| Both parents | 13403 (90.4) | 752 (91.7) | 2408 (90.1) | 4370 (91.2) | 3362 (89.2) | 2511 (90.6) | 10498 (90.6) | 592 (90.5) | 1917 (90.4) | 3266 (90.5) | 2646 (90.8) | 2077 (90.7) |
| One biological parent | 1126 (7.6) | 48 (5.9) | 202 (7.6) | 334 (7.0) | 321 (8.5) | 221 (8.0) | 880 (7.6) | 49 (7.5) | 159 (7.5) | 275 (7.6) | 220 (7.5) | 177 (7.7) |
| Without biological  parent | 296 (2.0) | 20 (2.4) | 62 (2.3) | 90 (1.9) | 85 (2.2) | 39 (1.4) | 208 (1.8) | 13 (2.0) | 45 (2.1) | 67 (1.9) | 48 (1.6) | 35 (2.1) |
| Attrition |  |  |  |  |  |  |  |  |  |  |  |  |
| No drop out | 10391 (70.1) | 597 (72.8) | 1958 (73.3) | 3388 (70.7) | 2583 (68.6) | 1865 (67.3) | 7788 (67.2) | 440 (67.3) | 1479 (69.7) | 2475 (68.6) | 1934 (66.4) | 1460 (63.8) |
| Drop out | 3226 (21.8) | 169 (20.6) | 596 (22.3) | 1103 (23.0) | 841 (22.3) | 517 (18.7) | 2487 (21.5) | 155 (23.7) | 472 (22.3) | 820 (22.7) | 634 (21.8) | 406 (17.7) |
| Death | 1208 (8.1) | 54 (6.6) | 118 (4.4) | 303 (6.3) | 344 (9.1) | 389 (14.0) | 1312 (11.3) | 59 (9.0) | 170 (8.0) | 314 (8.7) | 346 (11.9) | 423 (18.5) |
| *Covariates* |  |  |  |  |  |  |  |  |  |  |  |  |
| BMI, kg/m^2^ |  |  |  |  |  |  |  |  |  |  |  |  |
| ≤24.9 | 6129 (41.9) | 476 (58.7) | 1341 (50.8) | 2055 (43.3) | 1408 (37.9) | 849 (31.3) | 3492 (30.5) | 280 (43.3) | 751 (35.6) | 1041 (29.1) | 792 (27.5) | 628 (27.9) |
| 25.0–29.9 | 5701 (39.0) | 254 (31.3) | 910 (34.5) | 1814 (38.2) | 1535 (41.3) | 1188 (43.8) | 5937 (51.8) | 282 (43.6) | 1030 (48.9) | 1909 (53.4) | 1551 (53.9) | 1165 (51.7) |
| ≥30.0 | 2794 (19.1) | 81 (10.0) | 390 (14.8) | 874 (18.4) | 773 (20.8) | 676 (24.9) | 2024 (17.7) | 85 (13.1) | 326 (15.5) | 622 (17.4) | 532 (18.5) | 459 (20.4) |
| Smoking status |  |  |  |  |  |  |  |  |  |  |  |  |
| Never smoker | 5892 (66.7) | 261 (50.6) | 908 (56.1) | 1726 (62.5) | 1676 (72.3) | 1321 (81.7) | 2434 (35.7) | 162 (39.0) | 459 (35.2) | 724 (35.0) | 620 (35.1) | 469 (37.0) |
| Ex-smoker | 1521 (17.2) | 154 (29.8) | 410 (25.3) | 525 (19.0) | 302 (13.0) | 130 (8.0) | 2800 (41.0) | 164 (39.5) | 553 (42.3) | 848 (41.1) | 690 (39.1) | 545 (43.0) |
| Current smoker | 1420 (16.1) | 101 (19.6) | 300 (18.5) | 512 (18.5) | 341 (14.7) | 166 (10.3) | 1587 (23.3) | 89 (21.5) | 294 (22.5) | 494 (23.9) | 457 (25.9) | 253 (20.0) |
| No. of chronic conditions |  |  |  |  |  |  |  |  |  |  |  |  |
| < 2 | 8196 (55.3) | 557 (67.9) | 1705 (63.8) | 2842 (59.3) | 1944 (51.6) | 1148 (41.4) | 7165 (61.8) | 448 (68.5) | 1406 (66.3) | 2366 (65.6) | 1727 (59.3) | 1218 (53.2) |
| ≥ 2 | 6629 (44.7) | 263 (32.1) | 967 (36.2) | 1952 (40.7) | 1824 (48.4) | 1623 (58.6) | 4422 (38.2) | 206 (31.5) | 715 (33.7) | 1243 (34.4) | 1187 (40.7) | 1071 (46.8) |
| Physical activity |  |  |  |  |  |  |  |  |  |  |  |  |
| Low | 9989 (67.4) | 652 (79.6) | 2029 (76.0) | 3419 (71.4) | 2391 (63.5) | 1498 (54.1) | 8404 (72.5) | 517 (79.1) | 1658 (78.2) | 2742 (76.0) | 2027 (69.6) | 1460 (63.8) |
| High | 4825 (32.6) | 167 (20.4) | 642 (24.0) | 1370 (28.6) | 1376 (36.5) | 1270 (45.9) | 3181 (27.5) | 317 (20.9) | 462 (21.8) | 867 (24.0) | 887 (30.4) | 828 (36.2) |
|  |  |  |  |  |  |  |  |  |  |  |  |  |
| *Adult socioeconomic status* |  |  |  |  |  |  |  |  |  |  |  |  |
| Level of education |  |  |  |  |  |  |  |  |  |  |  |  |
| Low | 11849 (83.0) | 361 (45.5) | 1736 (67.8) | 3767 (81.7) | 3385 (93.0) | 2600 (97.3) | 8531 (76.8) | 240 (38.3) | 1214 (59.3) | 2594 (75.0) | 2440 (87.8) | 2043 (93.2) |
| High | 2428 (17.0) | 432 (54.5) | 824 (32.2) | 846 (18.3) | 255 (7.0) | 71 (2.7) | 2571 (23.2) | 386 (61.7) | 832 (40.7) | 865 (25.0) | 340 (12.2) | 148 (6.8) |
| Main occupation class |  |  |  |  |  |  |  |  |  |  |  |  |
| Low skill | 12282 (83.7) | 408 (50.2) | 1865 (70.2) | 3952 (82.8) | 3440 (92.3) | 2617 (96.7) | 7878 (69.1) | 197 (30.2) | 1047 (49.6) | 2337 (65.2) | 2323 (81.2) | 1974 (90.1) |
| High skill | 2393 (16.3) | 405 (49.8) | 793 (29.8) | 819 (17.2) | 286 (7.7) | 90 (3.3) | 3520 (30.9) | 455 (69.8) | 1065 (50.4) | 1245 (34.8) | 537 (18.8) | 218 (9.9) |
| Household income  (able to make ends meet) |  |  |  |  |  |  |  |  |  |  |  |  |
| Easily | 5170 (34.9) | 485 (59.3) | 1333 (49.9) | 1908 (39.9) | 998 (26.5) | 446 (16.1) | 4499 (38.9) | 421 (64.4) | 1159 (54.6) | 1597 (44.3) | 887 (30.5) | 435 (19.0) |
| Fairly easily | 4525 (30.6) | 226 (27.6) | 802 (30.0) | 1493 (31.2) | 1201 (31.9) | 803 (29.0) | 3602 (31.1) | 161 (24.6) | 638 (30.1) | 1169 (32.4) | 926 (31.8) | 708 (31.0) |
| With some difficulty | 3368 (22.8) | 87 (10.6) | 391 (14.6) | 977 (20.4) | 1008 (26.8) | 905 (32.7) | 2396 (20.7) | 61 (9.3) | 252 (11.9) | 625 (17.3) | 377 (24.7) | 740 (23.4) |
| With great difficulty | 1733 (11.7) | 20 (2.4) | 144 (5.4) | 402 (8.4) | 556 (14.8) | 611 (22.1) | 1081 (9.3) | 11 (1.7) | 72 (3.4) | 218 (6.0) | 718 (13.0) | 403 (17.6) |

*BMI* body mass index, *SD* standard deviation

Data are n (%) unless indicated
